# Assessing Consumer Preference for Overpackaging Solutions in E-Commerce

**DOI:** 10.3390/ijerph18157951

**Published:** 2021-07-27

**Authors:** Guojie Xie, Lijuan Huang, Chrysostomos Apostolidis, Zuqing Huang, Weiwei Cai, Guokai Li

**Affiliations:** 1 School of Management, Guangzhou University, Guangzhou 510006, China; 1111965003@e.gzhu.edu.cn (G.X.); hzq1210@gzhu.edu.cn (Z.H.); 2 Newcastle Business School, Northumbria University, Newcastle upon Tyne NE1 8ST, UK; c.apostolidis@northumbria.ac.uk; 3 Graduate School, Northern Arizona University, Flagstaff, AZ 86011, USA; wc358@nau.edu; 4 Department of Civil, Environmental and Architectural Engineering (DICEA), University of Padua, 35131 Padua, Italy; guokai.li@studenti.unipd.it

**Keywords:** electronic commerce, overpackaging, solutions, consumer preference, SmartPLS

## Abstract

The emergence of e-commerce and express delivery services has significantly transformed business operations and consumer shopping experience. However, the resulting problem of packaging waste, particularly from overpackaging, poses serious challenges to environmental sustainability and human health. Existing research has proposed many solutions from various perspectives, but very few have considered the acceptability and consumer preference for these proposals. Using the value co-creation (VCC) theory, we established a research model to explore consumer preferences for e-commerce overpackaging solutions. A survey of 632 online consumers in Guangzhou and Shenzhen was conducted, and data were analyzed using the SmartPLS software. The results show that establishing a recycling system, government policy, and consumers’ environmental awareness have a significant positive impact on consumer preference, while combined packaging has a significant negative impact. We also found that government policy plays an intermediary role in establishing a recycling system and consumer preference. Based on these findings, we recommend that enterprises establish and improve their packaging recycling systems and that e-commerce platforms provide alternative options to combined packaging. Also, the government should play a guiding and coordinating role for enterprises and consumers, and environmental awareness among consumers should actively be promoted.

## 1. Introduction

With the progress of communication technology and increasing ubiquity in mobile smartphone applications, e-commerce has become a crucial channel for consumers to shop online [1]. Analysts predict that e-commerce market coverage will grow by 25 percent by 2026 [2]. The COVID-19 pandemic has also prompted many brick-and-mortar stores to turn to online sales [3]. Over the years, consumers have become more supportive of this safe and convenient shopping option [4,5].

Recent data have shown that the development of e-commerce has facilitated the express delivery industry’s prosperity. For example, China’s express delivery volume reached 63 billion pieces in 2019, contributing more than 50% to the growth of global parcel volume [6]. According to the prediction of Joerss et al. [7], the express delivery volume in Germany and the United States will double in the next decade (till 2025), reaching an annual increase of about 5 billion and 25 billion, respectively. However, the rapid development of e-commerce has brought added pressure and challenges to environmental protection [1,8]. In particular, the overpackaging of products has become a major ecological concern [1,5,9]. The rapid growth of the express delivery industry has led to massive wastes and pollution concerns brought by overpackaging [1]. To protect products from damages during the distribution process and avoid negative comments from consumers, merchants often become guilty of overpackaging [1,10]. Many enterprises use packaging as part of their marketing strategy and invest in packaging features to enhance consumer experience [5]. These may then lead to the problem of overpackaging. In this paper, overpackaging in e-commerce is defined as the packaging that contains excessive consumable materials, high weight, large volume, extra high cost, and redundant decoration in the context of online shopping.

Overpackaging not only hinders environmental sustainability but also affects supply chain costs [11]. On the one hand, overpackaging results in increased energy consumption and carbon dioxide emissions [8,12]. Due to the use of degradation-resistant materials, overpackaging can endanger the health of users [13,14], pollute the land [15,16], and threaten the lives of Marine animals [1,17]. On the other hand, the increase in the product supply chain [11] and protective materials [9,18] can add costs to the supply chain.

Due to these adverse effects, numerous studies have explored solutions to overpackaging in e-commerce using comparative analysis (e.g., Pålsson et al. [19]; Zhao et al. [20]), literature reviews (e.g., Sílvia, et al. [5]; Meherishi et al. [11]), case studies (e.g., Gustavo et al. [21]), and model optimization (e.g., Brinker and Gündüz [22]; Dutta et al. [23]). However, previous studies have mainly proposed solutions from the perspectives of enterprises and governments, with only a few focusing on consumer preference or acceptance for these solutions (see Lu et al. [1] for a notable exception). Therefore, the current research aims to address this gap in the existing literature by answering the following question: which overpackaging solution in e-commerce do consumers prefer?

To answer this question, in this paper we adopt a value co-creation lens, and following a thorough literature review, we constructed a theoretical model focusing on how overpackaging in e-commerce solutions can affect consumer preferences. We modelled and tested consumer preferences for different overpackaging solutions (from the perspective of enterprises, government, and consumers) and explored the mediating role of government policies on and consumer awareness of environmental protection by means of structural equation modelling using the SmartPLS software. By adopting a VCC lens to explore consumer preferences for different overpackaging solutions, we aim to contribute to existing knowledge, theory, and practice in different ways. The first contribution of this paper to the existing literature is its focus on consumer preferences for different e-commerce overpackaging solutions, providing new insights for environmental protection, consumer satisfaction, and rapid marketization of solutions. In addition, we contribute to existing theory and literature on sustainable value co-creation by introducing the VCC theory as the research framework. The application of this theoretical framework helps to enrich related research on online shopping and sustainability and extends the framework to other disciplines (e.g., logistics and packaging design).

From a practical perspective, the identification of consumer preferences regarding the different e-commerce overpackaging solutions has the following benefits: (1) it can enable enterprises and governments to develop more sustainable solutions while still meeting consumer needs; (2) it can contribute to the rapid marketization and promotion of the solutions; (3) it is conducive to the improvement of the ecological environment and the reduction of packaging waste.

The remainder of the paper consists of five sections. Section 2 discusses the literature review. Section 3 analyzes the theoretical background, constructs the theoretical model, and presents the research hypotheses. Section 4 presents the research method and the empirical results. Section 5 discusses the theoretical implications and practical implications of our study and proposes some recommendations. Finally, Section 6 summarizes the main conclusions and discusses the limitations of the study.

## 2. Literature Review

### 2.1. The Emergence of the Overpackaging Problem in E-Commerce

E-commerce refers to exchanging goods and services between buyers and sellers through electronic media (e.g., the Internet and other digital platforms). Depending on the nature of the participants, e-commerce can be divided into four categories: business-to-business (B2B), business-to-government (B2G), business-to-consumer (B2C), and consumer-to-consumer (C2C) [9]. For this study, to enable a more extensive collection of data from a larger number of participants, we contextualize and investigate the problem of overpackaging, focusing on the B2C category.

Consumer satisfaction is a primary focus of e-commerce and serves as a critical link in the later stage of the supply chain [24]. Merchants have to ensure the quality of products and services being offered online. In addition, enterprises must also consider packaging and other logistics requirements to secure the product during shipping and handling [25]. In general, three tiers are used to distribute a product from merchants to consumers, namely primary packaging, secondary packaging, and tertiary packaging [5,19]. Primary packaging is used for containment, protection, and promotion and serves as the last piece of packaging between the consumer and the product. Secondary packaging, usually in large boxes or containers, is used to group multiple primary packaged goods. Tertiary packaging is used to aggregate the secondary packed boxes into larger containers for easy loading and unloading [5]. To meet marketing demands or improve customer experience, merchants may invest resources and add packaging design to support consumer satisfaction, loyalty, and intention to buy again [26].

However, efforts to enhance consumer shopping experience can cause environmental problems, such as added waste from overpackaging [20]. A typical e-commerce parcel may use multiple packaging materials, such as paper, envelopes, cardboard, plastic bags, woven bags, tape, and cushioning materials (e.g., bubble wrap, polystyrene foam, and bubble wrap) [9,18]. While many packaging materials, such as corrugated boxes, can be recycled or reused, most packaging waste ends up in municipal solid waste (MSW) streams destined for landfills or incinerators or get discarded without proper treatment [14]. Furthermore, Loon et al. [27] found that 100 g of corrugated board and filler materials (33 g in total) generate about 181 g of CO_2_-eq per product. The lack of an effective packaging recycling system [28] and consumer tendency to discard packaging materials [9] also contributes to increased waste and environmental pollution. Moreover, almost all plastic materials currently in use are non-biodegradable [14]. With the rapid growth of e-commerce in recent years, overpackaging has become a major source of pollution and global environmental concern [1,5,14,19,20].

### 2.2. Solutions for E-Commerce Overpackaging

Numerous studies have proposed solutions for e-commerce overpackaging based on an enterprises’ perspectives (merchants), governments, and consumers (e.g., Lu et al. [1]; Chueamuangphan et al. [9]; Meherishi et al. [11]; Duan et al. [14]).

#### 2.2.1. Enterprise Solutions

From the perspective of enterprises, recommendations and proposed solutions include using different packaging materials, developing a recycling system, combined packaging, and packaging redesign.

First, packaging materials had been estimated to account for 22 percent of the carbon impact of e-commerce [29]. Innovating packaging materials, reducing waste, and using biodegradable and reusable materials can effectively increase the packaging’s environmental performance [9]. For example, Sílvia et al. [5] and Suhas et al. [30] proposed using cellulose-based materials in disposable packaging. Found in woody and non-woody plants, cellulose is a natural, abundant, renewable material used in forestry and wood industries, agricultural practices, and industrial wastes [31,32] that has a shallow environmental impact [33].

The second aspect is the recycling system. The establishment of a recycling system is consistent with the circular economy principle and the concept of sustainable development [34], where manufacturers can reuse waste as secondary raw materials [35]. Research shows that the promotion of reuse and recycling is conducive to the transition from e-commerce to green commerce (e.g., Lu et al. [1]; Chueamuangphan et al. [9]). However, this would require enterprises to introduce advanced packaging recycling technologies (Dutta et al. [23]; Song et al. [36]) and find ways to effectively encourage consumers to participate in recycling programs (Chueamuangphan et al. [9]).

Third is combined packaging. Loon et al. [27] found that the size of the shopping basket is a crucial factor determining the sustainability of the e-commerce environment. Large suppliers distribute products from different supply points and divide large orders into several packages, which would require more packaging materials and produce more waste. Hence, determining the adequate container size and adopting an efficient packaging strategy are critical factors to save materials and transportation [22].

Finally, there is the option of redesigning the packaging. In many cases, the packaging of a single commodity may suffer from an unreasonable box design, waste of space, and excessive use of materials [37]. With the use of innovative packaging designs, the amount of waste, packaging volume, cost, and loss of goods can be significantly reduced [5]. This would also result in the packaging becoming easier to disassemble, more recyclable, and more sustainable [38].

#### 2.2.2. Government Solutions

At present, most government solutions have been aimed at merchants (e.g., e-commerce enterprises, delivery service providers). For example, the Chinese government instituted policies similar to extended producer liability. The aim is to encourage express service providers to recycle post-consumer packaging waste and include recyclability in industry standards for express packaging products [14]. Chueamuangphan et al. [9] argue that the government could make e-commerce companies responsible for collecting and managing packaging waste through a recycling plan or deposit return system. Other scholars have proposed creating incentives and penalty measures, levying commodity packaging fees, or imposing environmental protection taxes to encourage enterprises to actively tackle the problem of overpackaging (e.g., Song et al. [36]; Zhang et al. [39]).

#### 2.2.3. Consumer Solutions

From the consumers’ perspective, previous studies have mainly focused on environmental awareness and the quantity of items purchased in online transactions. Scholars (e.g., Duan et al. [14]; Deng [40]; Elgaaïed-Gambier [41]) agree that consumers should be cognizant of the environmental consequences of overpackaging and highlight the need to enhance their awareness for environmental protection. Consumers’ increased awareness of environmental protection would lead to changes in shopping behavior, especially in the number of items purchased in a single transaction. For example, Loon et al. [27] showed that consumers could effectively ameliorate the environmental impact of B2C e-commerce by increasing the number of goods they purchase in each session. Avoiding impulse single-product purchases and raising collective purchasing of goods can also be considered as positive steps towards environmental responsibility [9].

Table 1 presents some recommended overpackaging solutions and relevant references that could be used in analyzing and understanding overpackaging solutions in e-commerce. Previous studies have focused mainly on proposing various solutions and how these measures could help reduce waste, but have largely ignored the acceptability of such solutions to the consumer (see Lu et al. [1] for a notable exception). Solutions are more likely to achieve desired environmental benefits and be genuinely applied by the market if they are strongly accepted and received by consumers [1,42]. To further explore this aspect and address this current research gap, we employ the VCC concept to explore and better understand the importance of consumer preferences for various e-commerce overpackaging solutions.

## 3. Theoretical Model and Hypothesis

### 3.1. Value Co-Creation Theory

Several researchers have employed the concept of Value co-creation (VCC) to explain the important role of consumers as key stakeholders to support environmental sustainability and create sustainable value (e.g., Apostolidis et al. [43]). Strongly related to the Service-Dominant logic, VCC is mainly used to describe the collaboration between multiple stakeholders [44], including suppliers, business partners, allies, and customers, working together to create value for all of the members within the value creation system [45]. From the perspective of co-creation, suppliers and customers, conversely, are not on opposing sides but are partners that interact with each other to create value and new business opportunities [46]. In terms of customer value from the view of service-dominant logic, customers are always the co-creators of value [47]. In other words, in VCC, consumers play an active role in creating value by interacting with other stakeholders and accepting and utilizing the value propositions offered by enterprises and organizations [44]. This perspective, also known as value-in-use, suggests that value is not created by the enterprise and consumed by the customer, but it is co-created during use, as customers evaluate and determine the value of a proposition based on its use [48].

Since value is determined and created through usage, value can be considered a ‘preference’ experience, i.e., the outcome that customers receive as a result of the experience of using a product (or service) with the particular attributes, characteristics, or expected outcomes that they ‘value’ or prefer [49,50,51]. Therefore, from this point of view, consumer preferences play a pivotal role in the process of value co-creation since these preferences will eventually determine whether and how specific product features or attributes (e.g., recyclable packaging) can be part of the value proposition of the enterprise, and whether the consumer will use them to co-create value. Furthermore, if a supplier’s value proposition (e.g., different packaging material) leads to the acquisition or retention of customers, and these customers have better experience than with other suppliers due to the additional value-in-use of these features, the customer will typically develop a preference for that supplier and engage in repeat purchases. This means that value is co-created not only for the consumer but also for the producer, supplier, and other stakeholders. For instance, in the context of this research, suppliers may offer recyclable packaging for their products. However, it is only when consumers prefer this type of packaging and actually ‘use’ it (i.e., recycle it) that this feature creates value for the consumer, the business, and society in general. As such, consumer preferences can be considered an important factor influencing the value propositions, value-in-use, and the value co-creation process between different stakeholders.

In this paper, e-commerce enterprises, governments, and consumers are the participants and collaborators in overpackaging solutions in e-commerce. The solutions vary from different perspectives and offer different value propositions. Particularly, as demonstrated by the value-in-use view of VCC theory, it is necessary to evaluate and determine the more favored solutions by the market according to the particularity (i.e., preference) of consumers’ use. In fact, when consumers tend to accept an overpackaging solution, value co-creation is induced between the solution proposer and consumers. Therefore, the VCC theory is suitable for the purposes of this research.

### 3.2. Research Model Construction

**Figure 1:**
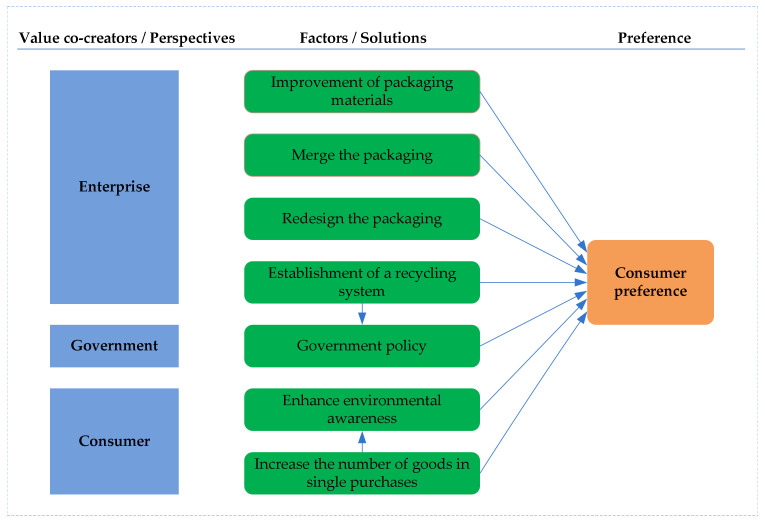
Consumer preference research model for e-commerce overpackaging solutions.

Based on the existing literature and drawing on VCC theory, we constructed a consumer preference model for e-commerce overpackaging solutions. The research model is shown in Figure 1. The model identifies seven factors that may affect consumer preferences. Based on the previous studies, we hypothesize that government policies play an intermediary role in the establishment of recycling systems [9,14], and that enhancing environmental awareness serves as an intermediary role in increasing the number of goods in each transaction [9,27].

### 3.3. Hypothesis

In this study, we adopt a VCC lens to evaluate consumer preferences for measures to reduce packaging waste. In line with the VCC theory, a number of different approaches and value propositions can be developed, as different stakeholders (e.g., suppliers, retailers, governments) can develop different value propositions or platforms, which consumers can utilize to co-create sustainable value. Consumer preferences for these solutions/value propositions are of key importance, since the attributes that the consumers prefer are generally the ones they use and continue using and, therefore, will eventually result in value creation. The measures assessed in this study are as follows: (1) improving packaging materials, (2) consolidation of packaging, (3) packaging redesign, (4) creation of a recycling system, (5) government policy, (6) increasing environmental awareness, and (7) increasing the number of goods in each purchase.

#### 3.3.1. Potential Preference for Enterprise Solutions

(1) Improvement of Packaging Materials

Previous studies have shown that improving packaging materials reduces pollution, minimizes harm to humans, and avoids product damage during transportation [31,40]. Better packaging material would ensure that the quality of delivered products improves consumers’ online shopping experience. In some cases, unnecessary tape and packaging can cause problems for consumers in unpacking and disposing of waste resources [11]. Improvements in packaging, such as antibacterial, transparent, water permeable, or oxygen permeable materials, can offer consumers more alternatives [52] so that they do not worry about damages to their purchased goods. Based on these arguments, we propose the following hypothesis:

**Hypothesis** **1** **(H1).** *The improvement of packaging materials is positively correlated with consumer preferences*.

(2) Merge the Packaging

For enterprises, combined packaging can help save packaging materials and decrease transportation costs [22]. However, for consumers, package bundling has both advantages and disadvantages. On the one hand, combining different packages can reduce the number of times consumers would have to pick up their packages. On the other hand, combining different goods would increase the time from the purchase to the receipt of the commodity. For some urgently needed goods, shipping and delivery delays are outright unacceptable. Lu et al. [1] found that consumers are very concerned about package delivery speeds in environmentally sustainable overpackaging solutions. In addition, combining products purchased on different platforms can be logistically challenging. Therefore, we propose the following hypothesis:

**Hypothesis** **2** **(H2).** *Consolidated packaging is negatively correlated with consumer preferences*.

(3) Redesigning the Packaging

The packaging redesign aims to optimize packaging space, reduce overuse of materials, and facilitate disassembly and recycling by consumers [5,37,38]. The optimization of packaging space can help prevent damage caused by squeezing or shaking during transport, and therefore improve consumer satisfaction [53]. Reinventing packaging that would be easier to disassemble and recycle can make waste disposal more accessible and convenient for consumers [14]. Hence, we propose the following hypothesis:

**Hypothesis** **3** **(H3).** *Packaging redesign is positively correlated with consumer preferences*.

(4) Establishment of a Recycling System

Creating a recycling system is an economical and eco-friendly solution to overpackaging [1,35]. For consumers, this is a pivotal part of supplementing online shopping services. Research shows that more than 11% of consumers habitually discard packaging materials directly after receiving express delivery [9]. A major reason for this is the absence of a recycling system for packaging materials [54]. For example, in China, JD Logistics and Cainiao Station have tried to provide tools for disassembling and packaging at express delivery pickup points and set up simple recycling schemes (e.g., providing packaging waste recycling bins) [55]. Such a scheme can encourage consumers to dispose of packaging waste at express points and prevent improper disposal. Also, a well-established recycling system helps to improve the external image of express delivery points so that consumers are not left with the impression that these areas are messy and full of waste. This is crucial to enhance the consumer experience at the end of shopping. Therefore, we propose the following hypothesis:

**Hypothesis** **4** **(H4).** *The establishment of a recycling system is positively correlated with consumer preferences*.

#### 3.3.2. Potential Preference for Government Solutions

(1) Government Policy

Government policy is reflected in the supervision, management, and coordination of enterprises on how they deal with the problem of overpackaging [36,39]. It also plays a crucial role in encouraging and guiding consumers to use green packaging. For example, the government has given e-commerce companies the responsibility of collecting and managing their packaging waste, including the development, coordination, and communication of recycling plans or deposit-return systems [9]. The Law of the People’s Republic of China on the Prevention and Control of Environmental Pollution by Solid Waste, revised in 2020, stipulates that at the national level, “producers and operators shall abide by mandatory standards to limit excessive packaging and avoid overpackaging” [56]. The government plays an active role in encouraging enterprises to explore ways to improve the recycling of packaging materials and guide consumers to actively participate in waste recycling programs [14]. According to VCC theory, when consumers accept the government’s value proposition, they will integrate resources and support each other to co-create value for both stakeholders [46]. Based on these arguments, we propose the following hypothesis:

**Hypothesis** **5** **(H5).** *The government policy is positively correlated with consumer preferences*.

(2) The Mediating effect of Government Policy Between the Establishment of Recycling System and Consumer Preferences

While the establishment of a recycling system and the introduction of government policies can directly affect consumer preferences for e-commerce overpackaging solutions, these measures can also result in some indirect effects. Supervision and management of government policies can help encourage and incentivize enterprises to develop more efficient recycling system and strategies [9,14]. Government policies can also be used to affect consumer preference for and acceptance of these recycling schemes. If enterprises lack government supervision and management, they may exhibit some resistance in investing resources in recycling programs, thus reducing government policies’ positive mediating effect. Accordingly, we propose the following hypothesis:

**Hypothesis** **6** **(H6).** *Government policies can mediate the relationship between the establishment of a recycling system and consumer preferences*.

#### 3.3.3. Potential Preference for Consumer Solutions

(1) Enhance Environmental Awareness

In general, environmentally conscious consumers demonstrate awareness and preferences for environmentally-friendly products and/or behaviors [1]. Consumer awareness and behavior for sustainable consumption can promote the transformation of e-commerce into green commerce [9]. Enhanced awareness for environmental protection is highly conducive for household and community waste management that would sort packaging materials for easier recycling [14]. Besides, consumers who are more environmentally aware tend to choose more eco-friendly packaging materials (e.g., biodegradable packaging), which boosts demands for sustainable packaging [57]. Consequently, we propose the following hypothesis:

**Hypothesis** **7** **(H7).** *Increasing environmental awareness is positively correlated with consumer preferences*.

(2) Increase the Number of Goods in Single Purchases

The reduction of complementary shopping behavior and maximization of the number of items purchased in each transaction would optimize packaging space and promote environmental sustainability in e-commerce [27]. For non-emergency goods, increasing the number of items per transaction, especially purchases on the same platform, would reduce the number of delivery and pickup times. For consumers, this collective purchase of individual products conforms with the concept of green packaging [9] and provides added convenience [1]. Therefore, we propose the following hypothesis:

**Hypothesis** **8** **(H8).** *Increasing the number of items per transaction is positively correlated with consumer preferences*.

(3) The Mediating Effect of Enhanced Environmental Awareness Between Items per Transaction and Consumer Preferences

While promoting environmental awareness and increasing the number of purchased goods per transaction can directly affect consumer preference, they can also indirectly affect consumer preference. Increasing environmental awareness can encourage consumers to buy more in bulk rather than single-item purchases [9]. In addition, consistent with the value-in-use perspective of VCC, enhancing consumers’ awareness of environmental protection can support sustainable value co-creation and avoid excessive pollutants, thus preventing possible land and sea pollution and damage to users’ health, and contributing to biodiversity. Based on these arguments, we propose the following hypothesis:

**Hypothesis** **9** **(H9).** *Increased environmental awareness will encourage consumers to purchase items in larger quantities*.

## 4. Research Methodology and Results

**Figure 2:**
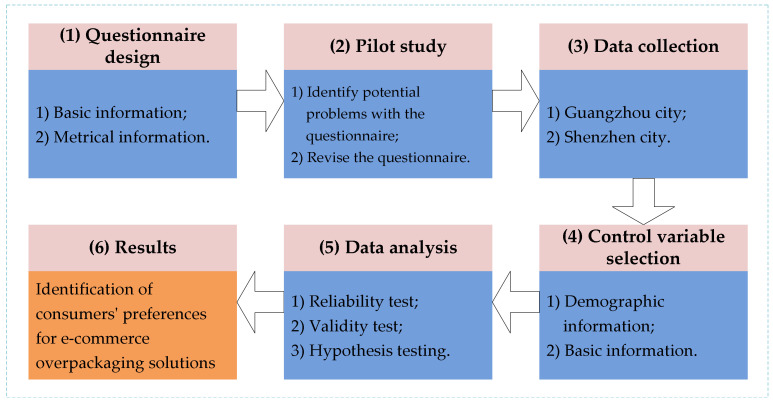
Research process.

The purpose of this paper is to investigate consumer preference for e-commerce overpackaging solutions adopting a VCC lens. Based on an extensive review of existing studies (e.g., Ye and Kankanhalli [58]; Huang et al. [59]), we developed the research process, as shown in Figure 2. The research process consists of the following steps: questionnaire design, pilot study, data collection, control variable selection, data analysis, and results.

### 4.1. Questionnaire Design

The questionnaire consists of two parts: (1) basic information of the respondents, and (2) measurement scales for the study variables. The respondent information includes gender, age, education level, occupation, city, online shopping experience, and attitudes towards overpackaging in e-commerce. The measurement scale contains eight latent variables, quantified using a five-level Likert scale, from “strongly disagree” to “strongly agree” [60]. The observation variables (presented in Table 2) corresponding to the latent variables were based on previous studies, and the final scale was formulated after consulting with numerous consumers.

### 4.2. Pilot Study

After developing the initial design for the questionnaire, a pretest was conducted to ensure the questionnaire’s validity and reliability and eliminate unclear expressions, repetitive sentences, and any other issues. The pretest lasted one week and was completed in December 2020. We surveyed 30 consumers with online shopping experience (15 in Shenzhen and 15 in Guangzhou) who were randomly selected and were willing to participate in the test. After answering the questionnaire, each of the respondents was interviewed for about five minutes. The contents of the post-survey interview include the following: (1) were there semantic ambiguities in any of the questions; (2) were there redundant items; and (3) do you have suggestions to improve the questionnaire. Based on the respondents’ feedback, we modified the language in some of the questions and deleted redundant questions for two variables (i.e., government policy and increasing items per transaction).

### 4.3. Data Collection

**Figure 3:**
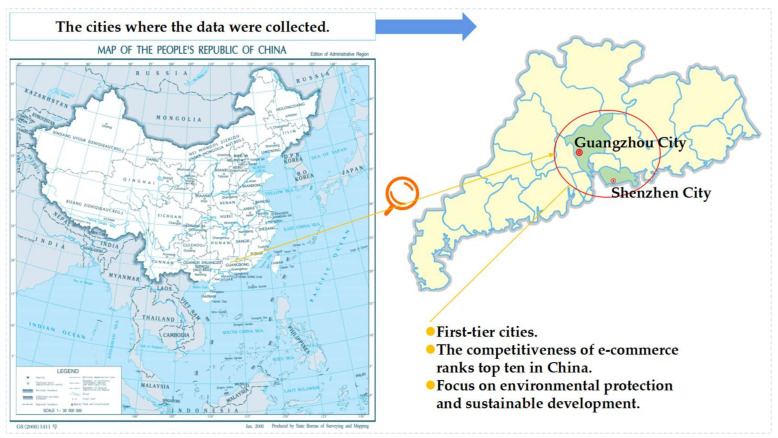
The cities where the data were collected.

The main survey was conducted in Guangzhou and Shenzhen in Guangdong Province, China (see Figure 3), which are considered first-tier cities. In 2019, Shenzhen and Guangzhou each had a permanent resident population of over 13 million [61,62]. The latest data released by the State Post Bureau shows that in the first half of 2019, the volume of express delivery in Guangzhou reached 2925.617 million pieces, ranking first among all Chinese cities. The volume of express delivery in Shenzhen reached 187.746 million pieces, ranking third among all Chinese cities [63]. According to the 2019 China E-Commerce Top 100 Data Report, Shenzhen and Guangzhou rank among the top 10 and are in the first echelon of e-commerce competitiveness in the country [64].

Having undergone significant economic reform and market liberalization, Shenzhen and Guangzhou pay considerable attention both to environmental protection and economic growth. Like many highly urbanized cities in China, these two cities have undertaken steps to integrate green and eco-sustainable strategies into their economic development. The research area selected in this study can be considered representative of other major economic cities in China.

The questionnaire survey was conducted online. The specific operations include the following: (1) sending the questionnaire link to social platforms (e.g., WeChat Moments, Weibo); (2) sending the questionnaire link to WeChat groups and QQ Groups, and then inviting those interested in participating; and (3) sending the questionnaire link to Taobao (a comprehensive online shopping platform in China) shopping groups. As a form of reward, the respondents were entered into a small lottery draw where they could win prizes, such as monetary rewards and shopping coupons. To ensure the reliability and authenticity of responses, we asked for some introductory and identification information at the beginning of the questionnaire, such as whether they have ever experienced online shopping, whether they have lived in Guangzhou or Shenzhen in the past year, and whether they are aware of the problems relating to overpackaging in e-commerce.

The average time for the respondent to finish the survey was about 5 min. The survey was conducted for two months, from December 2020 to January 2021, with 729 questionnaires received. After screening, 632 questionnaires were deemed valid, with an effective rate of 88.7%. Table 3 summarizes the results for the first part of the questionnaire (basic information).

As shown in Table 3, more women participated in the survey, accounting for 61%. Most respondents were between 20 and 35 years old, and their education level was relatively high. There was no significant difference in the sample size between Guangzhou and Shenzhen. All respondents have experienced shopping online, with 81% of respondents recognizing the overpackaging problem in e-commerce. Students, formal staff, and freelancers accounted for 74% of the respondents.

### 4.4. Control Variables

Control variables refer to all of the external and unrelated factors that affect the results, except the examined factors (independent variables) [65]. These unrelated variables are outside the scope of this paper and were not to be studied in this research [65]. Therefore, there is no need to test the effect of these control variables in the model. To minimize the impact of extraneous variables on the research results, based on the recommendations of Ye and Kankanhalli [58], Huang et al. [59], and Chang et al. [66], we used the respondent demographic information as control variables. These parameters include the consumers’ gender, age, education level, online shopping experience, occupation, and awareness of e-commerce overpackaging. Online shopping experience refers to whether consumers have bought goods through e-commerce platforms that need packaging and delivery. Understanding the problem of e-commerce overpackaging refers to whether the respondent thinks overpackaging is a problem in e-commerce.

### 4.5. Data Analysis

We used SmartPLS software (SmartPLS GmbH, Bönningstedt, Germany) to develop the structural equation model (SEM) for the empirical analysis. SEM has potential advantages over linear regression models and is the preferred method for analyzing path diagrams involving latent variables with multiple indicators [67]. SEM can integrate measured and hypothesized causal paths into an evaluation model [67]. Partial Least Squares (PLS) path modelling solves the measurement error problem by creating agents for latent variables suitable for empirical analysis and exploratory research of multistage structural equation models [67,68].

In the SmartPLS software, PLS-SEM can perform hypothesis verification on unobservable and challenging-to-measure latent variables [69], making it ideal for business research, behavioral science, statistics, and social sciences [59]. For example, Haverila and Haverila [70] used PLS-SEM and SmartPLS software to study customer perceptions of project management performance. Sabiu et al. [71] used SmartPLS-SEM to test the relationship between human resource management practices (recruitment and selection) and organizational performance. Numerous studies have shown SmartPLS-SEM can be applicable for studies in consumer preference for e-commerce overpackaging solutions.

#### 4.5.1. Reliability and Validity Test

**Figure A1:**
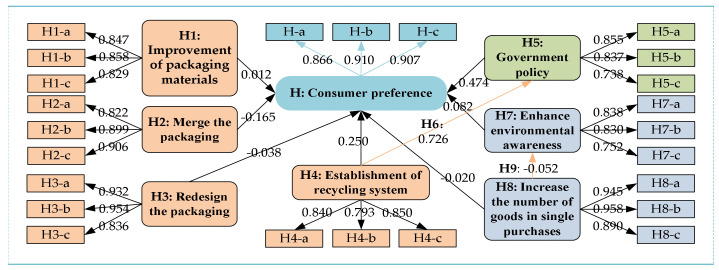
The model results, showing individual items.

We used SmartPLS software to build the PLS-SEM model (shown in the Appendix A, Figure A1) and tested the scale’s reliability and validity [72]. As shown in Table 4, the Cronbach’s alpha (CA) and Combinatorial Reliability (CR) of each latent variable achieved the relevant thresholds (>0.7). The external loading value in each observed variable in Figure A1 was greater than 0.7 (the lowest observed value is 0.738). The Average Variance Extracted (AVE) of the structure variables was greater than 0.5 (the lowest is 0.652) [73]. These results indicate that the scale and model have high reliability and validity and that the data have good convergence validity. As shown in Table 5, the square root of AVE was greater than the correlation coefficient with other latent variables, which suggests that the model has very good discriminant validity and that no multicollinearity exists among the latent variables [73].

To minimize the influence of common method bias (CMB), we used procedural control and statistical tests [74]. At the start of each survey questionnaire, we indicated that the questionnaire would be anonymous and that the data would be used only for academic research and not for any other purpose. Before conducting the actual survey, we conducted a pretest to find possible sources of confusion or error (e.g., respondents cannot understand some professional terms, incorrect statements, and incorrect logic of questions). We also used SPSS software and a Harman single factor test to conduct exploratory factor analysis for all observed variables (a total of 24) of the latent variables [74]. The results (see Table 6) show that no single factor could express most of the variability (>40%).Six factors (observed variables, not latent variables) had initial eigenvalues greater than one, and the explanatory power of the first factor was only 26.886%. Based on these results, common method bias was not considered an issue in this study.

#### 4.5.2. Hypothesis Testing

In the SmartPLS software, we analyzed the proposed research hypotheses using the PLS-SEM bootstrapping operation (shown in the Appendix A, Figure A1). As shown in Table 7, hypotheses H2, H4, H5, H6, and H7 passed the hypothesis tests (*p*-value ≤ 0.05) [73], while H1, H3, H8, and H9 were not verified (*p*-value > 0.05) [73]. The R^2^ (R-squared) of the PLS-SEM met the relevant thresholds, which indicates that the model has good explanatory capability [73]. Also, we have tested the overall fit of our model. The overall fit for the PLS-SEM was determined by SRMR and NFI [75]. A value of SRMR below 0.08 indicates that a PLS path model provides a sufficient fit of the empirical data [76]. Meanwhile, the use of the NFI usually is not recommended, as it systematically improves for more complex models [76]. This view is also echoed by [77]. In our study, the SRMR value of the PLS-SEM was 0.059, indicating that the PLS-SEM provides a good model fit [76].

### 4.6. Findings

#### 4.6.1. Significant Hypotheses

(1) The merging of packaging has a significant negative impact on consumer preferences, contrary to the findings of Lu et al. [1]. While combined packaging may reduce the number of times consumers pick up items, it may cause inconvenience to consumers. For example, combined packaging may prolong delivery times, and large volumes of goods may not be convenient for customer transportation and storage. Also, combining packaging for different goods may not always be advisable due to contamination risks or the mixing of smells. Consumers can be very particular about the delivery speed, the convenience of receiving goods, and the integrity of goods, which can be affected adversely by the merging of packaging.
(2) The results also show that creating a recycling system has a significant positive effect on consumer preference, supporting some previous studies (e.g., Lu et al., [1]; Chueamuangphan et al. [9]; Duan et al. [14]; Klemm et al. [31]). A major reason consumers discard packaging materials immediately after delivery is the lack of a (satisfactory) waste recycling system [54]. Establishing a recycling system will provide consumers with direct, convenient, and effective waste recycling solutions.
(3) Government policy was found to have a significant positive impact on consumer preference, a finding corroborating previous studies (e.g., Chueamuangphan et al. [9]; Duan et al. [14]; Song et al. [36]). Government policies can supervise and encourage enterprises to take actions on the overpackaging problem [39] and play an essential role in guiding consumers to actively tackle the problem of overpackaging solutions [14]. When companies or individuals commit serious violations of laws and regulations regarding overpackaging e-commerce solutions, consumers see government policy as an effective means of supervision and control.
(4) Enhancing environmental awareness has a significant positive impact on consumer preferences. This is consistent with the conclusions of Deng [40], Elgaaied-Gambier [24], and FoodBev [57]. Consumers with high environmental awareness are more inclined to select sustainable packaging [1] and be more cautious about how they discard packaging wastes. Environmental awareness prompts people to adopt more eco-friendly and sustainable choices to reduce overpackaging in e-commerce [57].
(5) Government policy plays an intermediary role in establishing a recycling system and consumer preference. Previous studies by Chueamuangphan et al. [9], Duan et al. [14], and Zhang et al. [39] have shown that the government can play a significant role in encouraging and supervising enterprises to participate in the formulation and implementation of overpackaging solutions. Our results confirm this view, indicating that government policies can amplify the effects of establishing recycling systems.

#### 4.6.2. Non-Significant Hypotheses

(1) Improving packaging materials had no significant effect on consumer preference. This is consistent with the results of Lu et al. [1] but contradicts the findings of Meherishi et al. [11], Klemm et al. [31], and Vilarinho [52]. The reason may be due to two aspects. First, consumers may worry that they will have to pay more for improved packaging materials [1]. Second, the difference in appearance between the improved and the ordinary packaging materials is not easily distinguishable.
(2) Packaging redesign also had no significant effect on consumer preference. This contradicts the findings of Duan et al. [14] and Williams et al. [53]. A possible reason is that aside from packaging redesign for damage prevention, other techniques, such as buffer foams and more efficient packaging methods, can be used to protect the products during transport [5]. While redesign can provide added protection, consumers may worry that added costs from the development and production of redesigned packaging would be passed to them, resulting in higher shipping and handling fees [21].
(3) Increasing the number of goods in single purchases had no significant effect on consumer preference. This is contrary to the results of Lu et al. [1], Chueamuangphan et al. [9], and Loon [27]. One possible explanation is that consumers’ shopping concerns are highly heterogeneous. Many who use online shopping want their purchases delivered immediately [78]. Consumers are even less likely to accept delays for some urgently needed items, such as perishable goods and medicine.
(4) There was no mediating effect of increasing environmental awareness on maximizing the number of items per transaction, which contradicts the findings of Chueamuangphan et al. [9]. One possible explanation is that while increased environmental awareness may lead to more eco-friendly behavior [1], maximizing items per transaction is just one of many green solutions which some consumers may not be able (or willing) to accept. For instance, this solution may not be acceptable to consumers with limited purchasing desire (or demands) and those that need to purchase various products on different platforms.

## 5. Discussion

Our results confirmed five of the nine research hypotheses concerning the impact of e-commerce overpackaging on consumer preferences, which can, in turn, affect the value co-creation process and the resulting value for consumers, suppliers, governments, and society in general. The establishment of a recycling system, government policy, and increasing environmental awareness have been positively correlated with consumer preference. Government policy was also found to have an intermediary role in establishing a recycling system and consumer preference. Merging of packaging was negatively correlated with consumer preference, which means this option was not viewed favorably by consumers. Except for the merging of packaging, the other confirmed hypotheses are consistent with the findings of previous studies. The results of the study have numerous theoretical and practical implications.

### 5.1. Theoretical Implications

This study explores consumer preferences for different overpackaging solutions that can affect e-commerce value propositions and the value created for different stakeholders. By adopting a VCC lens to explore the concept of overpackaging, this study links the concept of consumer preferences to sustainable value co-creation and explains how consumer preferences can influence the value-in-use for more sustainable products.This study also enriches the literature on e-commerce overpackaging solutions. The problem of overpackaging in e-commerce has been recognized and studied by the academic, public, and private sectors. While various solutions have been proposed from the perspectives of government (e.g., Chueamuangphan et al. [9]; Song et al. [36]; Zhang et al. [39]), enterprises (e.g., Sílvia et al. [5]; Dutta et al. [23]; Suhas et al. [30]) and consumers (e.g., Duan et al. [14]; Deng [40]; Elgaaïed-Gambier [41]), few have focused on consumer preferences. By focusing on the consumers’ psychological feelings and preferences, this paper provides new insights, which can help enterprises and governments formulate more effective solutions for overpackaging.

In order to contribute to existing literature and provide a more holistic understanding of the factors that can affect value co-creation from a consumer perspective, this study comprehensively evaluated consumer preference for overpackaging in three aspects: enterprises, government, and consumers. This approach varies from other studies, which generally studied overpackaging solutions through literature review (e.g., Sílvia et al. [5]), scaling experiments (e.g., Lu et al. [1]), secondary data analyses (e.g., Chueamuangphan et al. [9]) and case studies (e.g., Gustavo et al. [21]). By empirically studying consumer preferences, we were able to identify more acceptable solutions to consumers and demonstrated that government policy plays a mediating role between establishing a recycling system and consumer preferences.

Moreover, our study shows that VCC theory provides a scientific and reasonable framework for analyzing consumer preference for e-commerce overpackaging solutions. The application of this theoretical framework helps enrich related research on online shopping, and extends the framework to other disciplines (e.g., logistics, packaging design).

### 5.2. Practical Implications

From a practical perspective, our study provides enterprises and governments with recommendations on developing solutions more acceptable to consumers. It also provides measures and strategies that promote environmentally friendly behavior among consumers. The following are the recommendations we propose based on the results of this study.

First, enterprises should establish and improve their packaging recycling system. Our results show a significant positive correlation between establishing a recycling system and consumer preference. This means that consumers generally accept this solution. However, due to inadequate recycling systems, more than 11% of consumers habitually discard packaging materials immediately after delivery [9,54]. This solution offers consumers convenience in discarding packaging waste and creates a system for enterprises to collect them.

Second, the government should play a role in guiding and coordinating enterprises and consumers. Our study found that government policy has a significant positive correlation with consumer preference and plays an intermediary role in establishing a recycling system and consumer preference. The government can develop relevant policies to encourage and supervise enterprises in adapting effective overpackaging solutions [36,39]. It also plays an essential role in promoting environmental awareness among consumers and encouraging eco-friendly behaviors [14]. The government should act as a manager and friend for enterprises and consumers, providing much-needed support while also playing supervisory and coordinator roles.

Third, e-commerce platforms should provide consumers with the option of merging the packaging. Our study found a significant negative correlation between combined packaging and consumer preference. This is contrary to previous studies by Lu et al. [1]). As discussed, this strategy has a number of pros and cons (e.g., the advantage is that consumers can reduce the number of pickup times, but the disadvantage is that the delivery time of goods may be delayed). E-commerce platforms should seriously consider providing consumers with the option of combined packaging.

Finally, consumers should raise their awareness of environmental protection. Our study found a significant positive correlation between increased environmental awareness and consumer preference. To promote environmental awareness, E-commerce platforms can provide consumers with recommendations on eco-friendly options when shopping online. The government can strengthen the publicity to consumers in the reasonable selection of packaging materials, classification, and recycling of packaging waste. Consumers can raise awareness of environmental protection by understanding the recycling process of packaging waste and the threat of overpackaging to the environment and human health.

## 6. Conclusions

E-commerce has become an indispensable way of shopping, resulting in the rapid growth of express delivery services. However, the resulting e-commerce overpackaging poses many challenges to environmental sustainability and human health. If these challenges are not mitigated or addressed, they could lead to more serious environmental problems, undermining human health and high-quality economic development. Previous studies have proposed various solutions from the perspective of enterprises, governments, and consumers. However, there is a lack of empirical research on the acceptability or consumer preference for these solutions.

This study constructed a research framework based on the VCC theory. The results show that establishing a recycling system, government policy, and consumer awareness of environmental protection have significant positive correlations with consumer preference, while combined packaging has a significant negative impact. We also found that government policy plays an intermediary role in establishing a recycling system and consumer preference. Our findings help to enrich the literature on e-commerce overpackaging solutions.

Based on the findings of this study, we recommend that enterprises establish and improve their packaging recycling systems. The government should also help guide and coordinate enterprises and consumers, and e-commerce platforms should provide consumers with the option of combined packaging. Consumers’ environmental awareness should also be actively increased. These recommendations are based on consumer perception, which can help formulate more effective policies and strategies for dealing with the problem of e-commerce overpackaging.

However, there are some limitations to our study. Our investigation samples come from two first-tier cities in China: Guangzhou, and Shenzhen (economically developed regions). Different regions (such as cities in undeveloped regions) or more diverse countries may have disparate results. For example, cultural differences between countries may influence the results of the study. Therefore, future studies can analyze consumer preference in other areas and compare the results of our study. Second, the impact of differences in logistics infrastructure was not considered in this study. More materials may be required to protect packages in areas with less-developed logistical infrastructure. Future studies can evaluate if there are significant effects on consumer perception. Third, there is no denying that there are some solutions that we have not fully taken into account. In particular, with the progress of technology, the overpackaging solution has also been updated. In the future, we need to discuss consumer preference for emerging solutions as well. Fourth, the limitations inherent to online questionnaires may locally influence the results. In the future, we can consider more ways (e.g., web crawler technology, purchase research data from target companies) to collect data and compare the results. Finally, local culture may have a considerable influence on consumer preference for e-commerce overpackaging solutions. Subsequent studies can consider local culture as the respondents’ grouping basis and conduct a controlled study. We hold the opinion that attention must be paid to the environmental problems caused by e-commerce overpackaging. It requires us to propose more scientific and reasonable solutions based on different perspectives and verify whether these measures are accepted and recognized by most consumers.

## Figures and Tables

**Table 1:** E-commerce overpackaging solutions.

Perspectives	Solutions	References
Enterprise	Improve packaging materials	[5,9,30,33]
	Establishment of the recycling system	[1,9,23,35,36]
	Merge the packaging	[22,27]
	Redesign the packaging	[5,38]
Government	Encourage delivery service providers to recycle post-consumption packaging waste	[14]
	Encourage delivery service providers to recycle post-consumption packaging waste	[9]
	Reward and punishment measures will be formulated for enterprises, andcommodity packaging taxes and environmental protection taxes will be levied	[36,39]
Consumer	Enhance awareness of environmental protection	[14,40,41]
	Increase the number of goods in single purchases	[9,27]

**Table 2:** Items of constructs in the proposed model.

Variables	Items	References
Improvement of packaging materials (IoPM)	1a: I think the improvement of packing materials can alleviate excessive packing.	[11,14,31,52]
	1b: I prefer to improve packaging materials as an environmentally friendly solution.	
	1c: Improvements in packing materials will help protect our goods during transit.	
Merge the packaging (MTP)	2a: I think merging the packaging will increase the package’s waiting time.	[1]
	2b: I hope the goods I purchased will be shipped as soon as possible.	
	2c: I think merging the packaging will reduce express delivery speed.	
Redesign the packaging (RTP)	3a: I think the packing redesign helps prevent damage caused by pressing or shaking in transit.	[5,14,37,53]
	3b: I think redesigning the packaging is convenient for consumers to disassemble the packaging box.	
	3c: I think the packaging redesign makes it easier for consumers to recycle the boxes.	
Establishment of recycling system (EoRS)	4a: I think the current recycling system is not well established.	[1,9,35,54]
	4b: I think setting up a recycling system is an environmentally friendly solution.	
	4c: I think a recycling system is an economical solution.	
Government policy (GP)	5a: I believe the government policy has a good role in supervising enterprises to deal with the problem of overpackaging.	[9,14]
	5b: I believe the government policy has a good management effect on enterprises dealing with overpackaging.	
	5c: I believe the government policy has played a role in encouraging and guiding consumers to use green packaging and decrement packaging.	
Enhance environmental awareness (EEA)	7a: I like to choose environmentally friendly packaging materials.	[1,9,14,57]
	7b: I prefer to create environmentally friendly e-commerce overpackaging solutions.	
	7c: I would like to see more eco-friendly behavior in e-commerce overpackaging solutions.	
Increase the number of goods in single purchases (ItNoGiSP)	8a: I have fewer emergency online purchases.	[1,9,27]
	8b: I like to make a shopping list at the beginning of every online shopping trip.	
	8c: I think increasing the number of single purchases can reduce the number of times and save the time of picking up items.	
Consumer preference (CP)	a: I think the e-commerce overpackaging solution implemented by enterprises should suit our needs.	[1,5,9,14]
	b: I think the government policy implementation on e-commerce overpackaging solutions needs to consider consumer preference.	
	c: I think it is essential for consumers to consider their own opinions when choosing an overpackaging e-commerce solution.	

**Table 3:** Descriptive statistics of the respondents’ necessary information.

Category	Item	Ratio
Gender	Male	39%
	Female	61%
Age	<20	15%
	20–25	32%
	26–30	21%
	31–35	16%
	36–40	12%
	>40	4%
Education level	≤Middle School	5%
	High school	26%
	Junior college	32%
	≥Bachelors	37%
Cities	Guangzhou	52%
	Shenzhen	48%
Online shopping experience	Yes	100%
	No	0%
Whether know the issue of e-commerce overpackaging	Yes	19%
	No	81%
Occupation	Formal staff	22%
	Entrepreneurs	7%
	Students	35%
	Freelancer	17%
	Unemployed	6%
	Other	13%

**Table 4:** Results of reliability and validity tests.

Variables	CA	CR	AVE
IoPM	0.800	0.882	0.714
MtP	0.849	0.909	0.769
RtP	0.902	0.934	0.826
EoRS	0.771	0.868	0.686
GP	0.739	0.852	0.659
EEA	0.732	0.849	0.652
ItNoGiSP	0.925	0.951	0.867
CP	0.875	0.923	0.800

Note: IoPM, improvement of packaging materials; MtP, merge the packaging; RtP, redesign the packaging; EoRS, establishment of recycling system; GP, government policy; EEA, enhance environmental awareness; ItNoGiSP, increase the number of goods in single purchases; CP, consumer preference.

**Table 5:** Average Variance Extracted (AVE) square root and factor correlation coefficient.

Variables	H1	H2	H3	H4	H5	H7	H8	H
IoPM	0.845							
MtP	−0.005	0.877						
RtP	0.156	0.121	0.909					
EoRS	0.316	−0.133	0.140	0.828				
GP	0.328	−0.283	0.129	0.726	0.812			
EEA	0.420	0.024	0.318	0.383	0.427	0.807		
ItNoGiSP	0.025	0.029	−0.024	−0.080	−0.062	−0.052	0.931	
CP	0.275	−0.335	0.066	0.648	0.737	0.370	−0.077	0.895

Note: Diagonal elements are the square root of Average Variance Extracted (AVE).

**Table 6:** Total variance explained.

Component	Initial Eigen Values			Extraction Sums of Squared Loadings		
	Total	% of Variance	Cumulative %	Total	% of Variance	Cumulative %
1	6.453	26.886	26.886	6.453	26.886	26.886
2	3.162	13.175	40.061	3.162	13.175	40.061
3	2.646	11.025	51.087	2.646	11.025	51.087
4	1.919	7.997	59.084	1.919	7.997	59.084
5	1.662	6.926	66.010	1.662	6.926	66.010
6	1.094	4.557	70.566	1.094	4.557	70.566
7	0.920	3.831	74.398			

**Table 7:** Results of hypotheses testing.

Hypothesis	Path	Path Coefficient	t-Value	*p*-Value	Hypothesis Supported?
H1	IoPM→CP	0.012	0.395	0.693	N
H2	MtP→CP	−0.165	5.535	0.000	Y
H3	RtP→CP	−0.038	1.800	0.072	N
H4	EoRS→CP	0.250	6.558	0.000	Y
H5	GP→CP	0.474	10.229	0.000	Y
H6	EoRS→GP	0.726	32.333	0.000	Y
H7	EEA→CP	0.082	2.433	0.015	Y
H8	ItNoGiSP→CP	−0.020	0.722	0.471	N
H9	ItNoGiSP→EEA	−0.052	1.230	0.219	N

## Data Availability

We would be happy to share the research data in this paper with interested scholars if necessary. We also hope to have a deeper discussion with scholars on the operation of SmartPLS software. If interested scholars have data requirements, please contact the first author by email (1111965003@e.gzhu.edu.cn). Looking forward to meeting more experts and scholars in the same field.
